# Association of modified cytosines and the methylated DNA-binding protein MeCP2 with distinctive structural domains of lampbrush chromatin

**DOI:** 10.1007/s10577-012-9324-x

**Published:** 2012-11-14

**Authors:** Garry T. Morgan, Peter Jones, Michel Bellini

**Affiliations:** 1 Centre for Genetics and Genomics, School of Biology, University of Nottingham, Queens Medical Centre, Nottingham, NG7 2UH UK; 2 Boston Biomedical Research Institute, 64 Grove Street, Watertown, MA 02472 USA; 3 Department of Cell and Developmental Biology, University of Illinois, 601 S. Goodwin Avenue, Urbana, IL 61801 USA

**Keywords:** Lampbrush chromosomes, Methylation, 5-hydroxymethylcytosine, Oocyte, Transcription unit

## Abstract

We have investigated the association of DNA methylation and proteins interpreting methylation state with the distinctive closed and open chromatin structural domains that are directly observable in the lampbrush chromosomes (LBCs) of amphibian oocytes. To establish the distribution in LBCs of MeCP2, one of the key proteins binding 5-methylcytosine-modified DNA (5mC), we expressed HA-tagged MeCP2 constructs in *Xenopus laevis* oocytes. Full-length MeCP2 was predominantly targeted to the closed, transcriptionally inactive chromomere domains in a pattern proportional to chromomeric DNA density and consistent with a global role in determining chromatin state. A minor fraction of HA-MeCP2 was also found to associate with a distinctive structural domain, namely a short region at the bases of some of the extended lateral loops. Expression in oocytes of deleted constructs and of point mutants derived from Rett syndrome patients demonstrated that the association of MeCP2 with LBCs was determined by its 5mC-binding domain. We also examined more directly the distribution of 5mC by immunostaining *Xenopus* and axolotl LBCs and confirmed the pattern suggested by MeCP2 targeting of intense staining of the chromomeres and of some loop bases. In addition, we found in the longer loops of axolotl LBCs that short interstitial regions could also be clearly stained for 5mC. These 5mC regions corresponded precisely to unusual segments of active transcription units from which RNA polymerase II (pol II) and nascent transcripts were simultaneously absent. We also examined by immunostaining the distribution in lampbrush chromatin of the oxidized 5mC derivative, 5-hydroxymethylcytosine (5hmC). Although in general, the pattern resembled that obtained for 5mC, one antibody against 5hmC produced intense staining of restricted chromosomal foci. These foci corresponded to a third type of lampbrush chromatin domain, the transcriptionally active but less extended structures formed by clusters of genes transcribed by pol III. This raises the possibility that 5hmC may play a role in establishing the distinctive patterns of gene repression and activation that characterize specific pol III-transcribed gene families in amphibian genomes.

## Introduction

Lampbrush chromosomes (LBCs; see http://www.projects.exeter.ac.uk/lampbrush) essentially comprise a transcriptionally hyperactive form of chromatin in which visible DNA loops bearing arrays of nascent transcripts extend from a chromosome backbone composed of relatively inactive and compact domains, the chromomeres (reviewed by Callan 1986; Morgan 2002; see Macgregor *ibid*.). Amongst other attributes therefore, LBCs have the useful feature of enabling morphologically discrete chromatin structural domains to be directly observed by light microscopy in the context of individual, identifiable chromosomes. The distinctiveness of lampbrush chromatin is usually interpreted as arising from adaptations associated with the transcriptional hyperactivity required for the construction by amphibian oocytes of a large “pre-embryo” within a single cell (Davidson 1986). However, it also seems likely that lampbrush chromatin possesses characteristics defined by the unique functions of gametes in particular and of germ cells in general, such as meiosis and the acquisition of totipotency. In the latter context, it is significant that amphibian oocytes can profoundly modify the chromatin of transferred somatic nuclei and reprogramme the patterns of gene activity of differentiated cells to more embryonic or stem-cell like states (Jullien et al. 2011). It may be therefore, that lampbrush chromatin has distinctive properties that uniquely reflect the dominant reprogramming potential of its natural environment.

Previous studies have identified some key properties of lampbrush chromatin such as the presence of particular modified core histones (Sommerville et al. 1993; Gaginskaya et al. 2009; Krasikova et al. 2009) and cohesin subunits (Austin et al. 2009), as well as the highly dynamic nature of the association of these proteins with LBCs (Stewart et al. 2006; Austin et al. 2009). However, lampbrush chromatin also appears distinctive with respect to the absence of typical chromatin components such as topoisomerase II (Hock et al. 1996), HMGN proteins (Korner et al. 2003) and condensin subunits (Beenders et al. 2003). Particularly intriguing is the apparent absence from lampbrush chromatin of H1 linker histones (Hock et al. 1993), given the many and varied roles that linker histones have in the structure and function of chromatin (Bustin et al. 2005). Reduced total levels of linker histones are also found in certain somatic cell types, notably mammalian neuronal cells and pluripotent embryonic stem cells (Woodcock et al. 2006). In the former case, recent data suggest that H1 histone may be replaced globally as a chromatin structural protein and general transcriptional repressor by the nuclear protein MeCP2 (Skene et al. 2010).

MeCP2 was first identified (Lewis et al. 1992) by its affinity for DNA containing 5-methylcytosine (5mC), the predominant modified base of vertebrate genomes. Methylation of DNA has long been associated with transcriptional repression and inactive chromatin as well as with the provision of a key mammalian epigenetic system for gene silencing (Klose and Bird 2006). At a chromosomal level, for instance, MeCP2 has been shown to be associated with highly methylated DNA in the pericentromeric regions of mouse metaphase chromosomes (Lewis et al. 1992). Recent genome-scale mapping of methylation has suggested that 5mC may also be found in other contexts, including in gene bodies (Jones 2012). In order to assess whether MeCP2 could be playing a structural role in any of the various distinctive morphological domains of lampbrush chromatin, we have investigated the targeting to LBCs of human MeCP2 constructs expressed in *Xenopus laevis* oocytes. Since the pattern of MeCP2 localization would be expected also to match the distribution of 5mC in lampbrush chromatin, we have investigated the latter in parallel. We have used the well-characterized antibodies now available to examine by immunostaining the LBCs of *Xenopus* and/or *Ambystoma mexicanum* (axolotl) with regard to the distribution of both 5mC and the oxidized 5mC derivative, 5-hydroxymethylcytosine (5hmC), which has recently been shown to exist at high levels in certain cell types (Kriaucionis and Heintz 2009; Tahiliani et al. 2009). We present evidence that MeCP2, 5mC, and 5hmC can all be associated with transcriptionally active structural domains of LBCs as well as with compact, transcriptionally inactive ones.

## Materials and methods

### Expression of HA-MeCP2 and mutants in *Xenopus* oocytes

A short sequence coding for the HA tag (YPYDVPDYA) was added in frame at the 5′ end of the open reading frame (ORF) coding for the *X. laevis* MeCP2. A short 5′ untranslated region sequence, routinely used in our lab for strong expression in frog oocytes (TGAGCCAGAACAATG) was then added by PCR immediately upstream of the HA tag. The resulting MeCP2-HA ORF was then cloned into the pCR®-Blunt II-TOPO® vector (Invitrogen, Carlsbad CA). The three MeCP2 deletion mutants were obtained by polymerase chain reaction (PCR), using the high fidelity Deep Vent_R_® DNA Polymerase (New England BioLabs, Ipswich, MA) and the MeCP2-HA cDNA as a template. In addition, ∆C203-486 and MBD received an SV40 NLS (CCA AAG AAG AAG CGA AAG CTG) in their 3′ end to ensure that the corresponding proteins would enter the nucleus. The amplified DNA fragments were cloned into the pCR®-Blunt II-TOPO® vector (Invitrogen, Carlsbad CA), which contains an SP6 and a T7 promoter localized upstream and downstream of the multiple cloning site, respectively. In vitro transcriptions were performed as described in Beenders et al. 2007. Template DNAs were obtained by linearizing the clones described above downstream of their respective ORF, and either SP6 or T7 polymerases, as required, were used to synthesize capped, sense-strand RNAs. Newly made RNAs were phenol/chloroform extracted, precipitated with ethanol, and resuspended in water. Their concentration was adjusted to ∼1 mg/mL. RNAs were microinjected into the cytoplasm of stage IV–V oocytes under a dissecting microscope (S6 Leica), using a nanoject II (Drummond, Broomal PA) and glass pipettes prepared using a horizontal pipette puller P-97 (Sutter Instrument, Novato CA). Injections had a constant volume of 30 nL (30 ng of RNA).

### Preparation and immunostaining of oocyte nuclear spreads

Oocyte nuclei (germinal vesicles; GVs) were manually dissected in GV isolation medium (83 mM KCl, 17 mM NaCl, 6.5 mM Na_2_HPO_4_, 3.5 mM KH_2_PO_4_, 1 mM MgCl_2_, 1 mM DTT; pH 7.0–7.2). Spread preparations of *X. laevis* GV contents were made using the procedure developed by Gall (Gall 1998; Gall and Wu 2010) and a slightly modified procedure used for axolotl GVs (Morgan 2008; see http://www.projects.exeter.ac.uk/lampbrush). After centrifugation to attach the GV contents to a microscope slide, preparations were fixed for a minimum of 15 min and a maximum of 1 h in 2 % paraformaldehyde made up in phosphate-buffered saline (PBS, 137 mM NaCl; 2.7 mM KCl; 10.2 mM Na_2_HPO_4_; 1.8 mM KH_2_PO_4_; pH 7.4) containing 1 mM MgCl_2_.

Prior to staining with primary antibodies directed against proteins, fixed preparations were rinsed in PBS and blocked by incubation in 10 % foetal calf serum in PBS for 30 min. The spreads were then incubated for 1 h at room temperature with primary antibodies, rinsed briefly with 10 % foetal calf serum and then incubated for 1 h with secondary antibodies diluted in PBS. Preparations were stained with DAPI (0.5 μg/ml in PBS) and mounted in 50 % glycerol/PBS. When co-staining with antibodies against modified cytosine was required, staining with DAPI and anti-protein antibodies, and sometimes imaging, were carried out prior to the denaturation/depurination pre-treatment used for staining with 5mC and 5hmC antibodies. The latter involved incubating preparations in 2 M HCl at 37 °C for 1 h, followed by four washes in PBS. Preparations were then immunostained as above for modified cytosines.

Primary antibodies were diluted in 10 % foetal calf serum as follows: mAb H5 (Warren et al. 1992) culture supernatant 1:50 dilution; α-RPC15 (Murphy et al. 2002) 1:500; mAb No34 (Murphy et al. 2002) 1:500; α-5mC mAb 33D3 (Active Motif) 1:200; α-5hmC mAb 59.1 (Active Motif) 1:4000; α-5hmC pAb (Active Motif 39769) 1:4000. Secondary antibodies, used at dilutions of 1–5 μg/ml, were Alexa 488-conjugated goat anti-mouse IgG, Alexa 594-conjugated goat anti-mouse IgM or goat anti-rabbit IgG (all Molecular Probes) and Cy2-conjugated goat anti-mouse Fc (gamma) fragment (Jackson Immunoresearch Laboratories). Phase contrast, DIC and fluorescence observations were made with an Olympus BX-60 microscope as described previously (Smith et al. 2003). Images were captured with a Princeton Instruments digital CCD camera (Roper Scientific) and processed using IPLab imaging software and Adobe Photoshop and Illustrator (Adobe Systems Inc).

### Immunoblotting

Cytoplasms and nuclei were hand-isolated and homogenized in GV isolation medium. Crude extracts were centrifuged at 22,000×*g* at 4 °C for 10 min. Clarified extracts were collected and fractionated on 5–15 % polyacrylamide/SDS gradient gels, using the Mini-PROTEAN 3 Electrophoresis System (Bio-Rad, Hercules, CA). Proteins were transferred to Immobilon membranes (Millipore, Bedford, MA) for 2 h at 40 V in Tris–glycine buffer containing 20 % methanol. Membranes were blocked in 5 % dry milk–PBS for 1 h at room temperature and then incubated for 1 h at room temperature in PBS–Tween (PBST) containing the anti-HA antibody mAb 3 F10 at the concentration of 20 ng/mL. Membranes were washed 3× with 1× PBS containing 0.05 % of Tween 20 (PBS2T) and incubated with the secondary antibody in PBST for 1 h at room temperature. After three washes with PBS2T, detection was performed using the ECF™ kit (GE Healthcare, Fairfield CT) and the FLA3000 fluoroimager (Fuji Medical Systems, Stamford CT).

## Results

### Targeting of MeCP2 to *Xenopus* lampbrush chromosomes

**Fig. 1:**
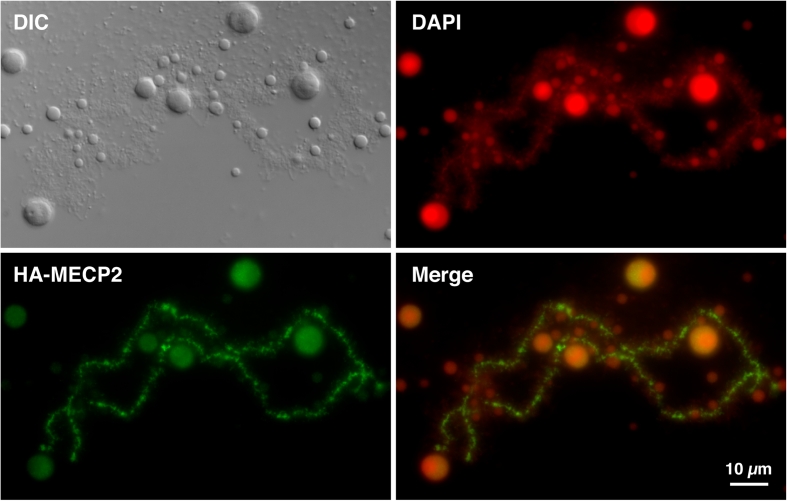
Chromosomal association of newly expressed HA-MeCP2. Newly made HA-MeCP2 in stage IV–V *Xenopus* oocytes targets the chromomeric regions of LBCs. DAPI staining (in *red*) shows that the chromosomal association of MeCP2 is proportional to the DNA density. While many lateral loops are present (they are readily visible by DIC), they appear to be devoid of HA-MeCP2. However, very short HA-MeCP2 positive lateral projections are readily visible and are likely to correspond to the bases of many loops. HA-MeCP2 also associates with nucleoli, but at a low concentration and primarily with the granular region

**Fig. 2:**
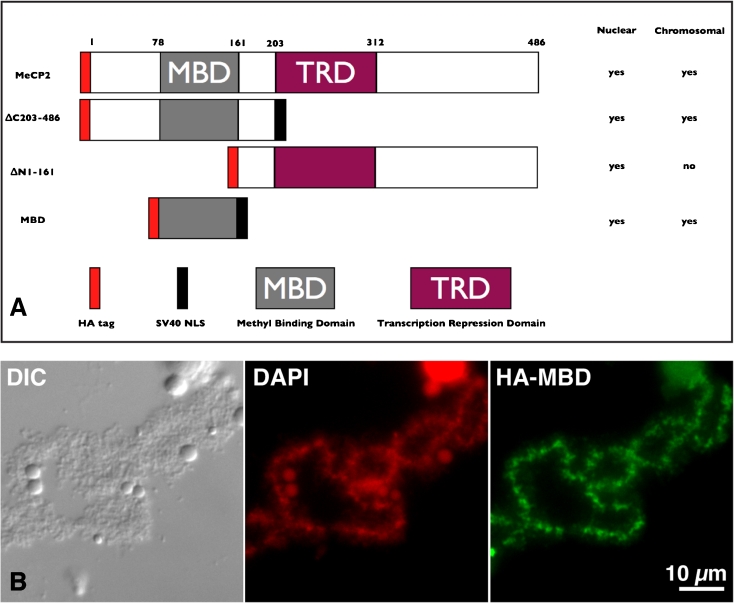
The MBD is necessary and sufficient for chromosomal association of MeCP2. **a** Schematic representation of three deletions of MeCP2. All truncated forms received a HA tag at the N-terminus. In addition, both ∆C203-486 and MBD were fused to a SV40 type NLS to promote efficient nuclear targeting (∆N1-161 already contains a NLS). Deletions were expressed in stage IV–V *Xenopus* oocytes and a summary of their respective subcellular distributions is provided. **b** DIC and corresponding fluorescent micrographs of the HA-MBD deletion. Just like wild-type HA-MeCP2, the newly made protein HA-MBD primarily targets the chromomeric regions of LBCs

**Fig. 3:**
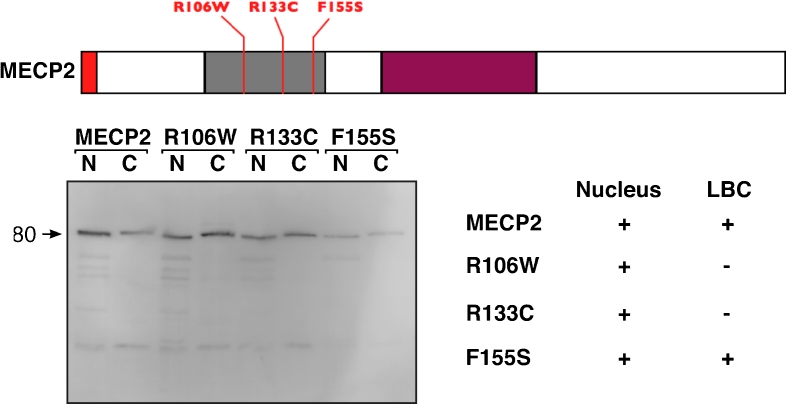
Expression of Human MeCP2 and three single substitution mutants. HA-tagged human MeCP2 was expressed in stage IV–V *Xenopus* oocytes. All proteins were expressed to comparable levels and recruited to the nucleus. Wild-type MeCP2 and, surprisingly, F155S mutant were the only two proteins able to associate with the chromomeric regions of LBCs

In many cell biology textbooks, LBC loops are usually discussed in support of models in which the chromatin of somatic nuclei is organized into numerous long-range loop domains. It is important to acknowledge, however, that these two types of loops are different in nature, as the LBC loops correspond to hyperactive pol II transcriptional sites rather than large genomic loop domains. Nevertheless, since LBCs offer a direct visualization of the “open” (lateral loops) and “closed” (LBC axes) states of chromatin domains, we investigated here the chromosomal distribution pattern of MeCP2, because it has previously been implicated in chromatin loop organization and more recently in transcription. Capped, sense-strand transcripts coding for HA-MeCP2 were produced in vitro and injected into the cytoplasm of stage IV–V *Xenopus* oocytes. Nuclei were isolated 18 h later and the intra-nuclear distribution of newly made HA-MeCP2 was analysed using the anti-HA antibody mAb 3F10. We found that newly made HA-MeCP2 is imported to the nucleus where it rapidly associates with LBCs and to a less extent with nucleoli (Fig. 1). Interestingly, chromosomal HA-MeCP2 predominantly displays a chromomeric pattern, which is proportional to DNA density as defined here by DAPI staining (Fig. 1). MeCP2 is a protein that displays two well-characterized functional domains, a methyl C binding domain (MBD) and transcription repression domain (TRD). Several truncated forms of MeCP2 were then generated and expressed in oocytes to assess the role of these domains in the intra-nuclear distribution of MeCP2 (Fig. 2). We found that MeCP2 lacking the MBD (∆N1-161) fails to target LBCs, while TRD is dispensable since ∆C203-486 associates with LBCs as well as the wild-type MeCP2. In fact, Fig. 3 shows that the MBD alone is able to produce a chromomeric pattern that is indistinguishable from the wild-type MeCP2 pattern. To further test the role of the MBD in establishing the chromosomal distribution of HA-MeCP2, we expressed in stage IV–V oocytes three distinct human MeCP2 proteins that each possess an MBD carrying a different point mutation (abbreviated as R106W, R133C and F155S, respectively). These mutations are often encountered in Rett syndrome patients and were previously shown to inhibit the binding of MeCP2 to methylated DNA in vitro (Ballestar et al. 2000). We found that although all three mutants are still recruited to the nucleus, two of these mutants, R106W and R133C, are unable to associate with LBCs (Fig. 3). Together, these data demonstrate the critical importance of the MBD for the association of MeCP2 with LBCs. In addition, since the in vitro DNA-binding activity of MBD requires the presence of methylated cytosines within the target sequence, our data strongly suggest that the newly expressed HA-MeCP2 binds methylated DNA regions of LBCs.

**Fig. 4:**
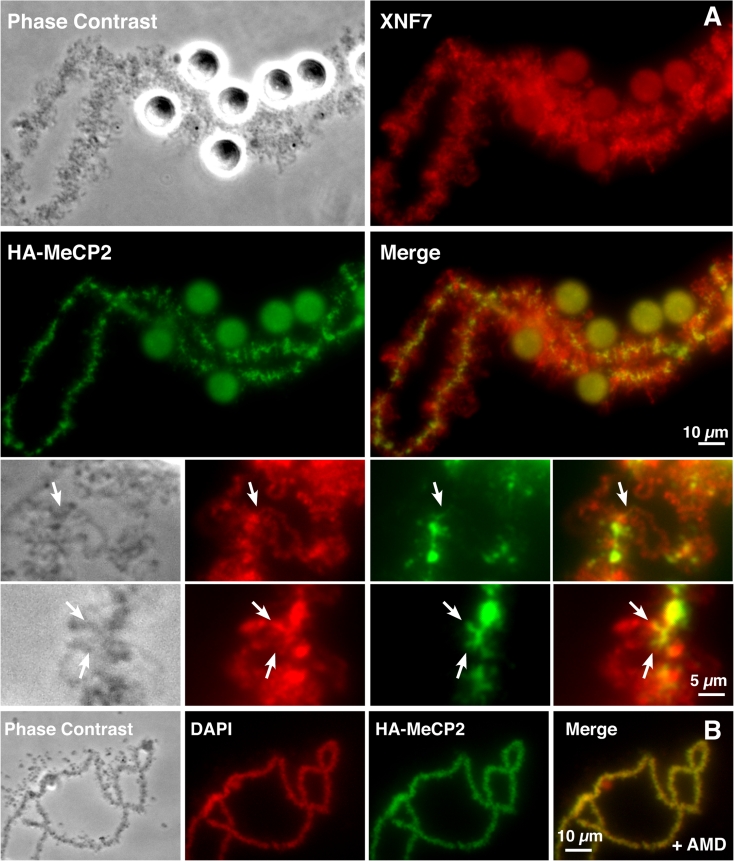
HA-MeCP2 targets the bases of loops transcribed by RNA pol II. **a** DIC and corresponding fluorescent micrographs of nuclear spreads where HA-MeCP2 (*green*) and NF7 (*red*) were co-localized. At a lower magnification, NF7 and HA-MeCP2 appear segregated into two distinct chromosomal domains: NF7 associates with the RNP matrix of most lateral loops, while HA-MeCP2 is primarily associated with chromomeres. Magnified views, however, reveal that HA-MeCP2 is also found at the base of some loops (*arrows*). **b** These short lateral projections of HA-MeCP2 disappear when loops retract upon treatment with AMD but the chromomeric distribution persists

While the majority of chromosomal HA-MeCP2 is associated with the axial chromomeres, there is also evidence of a small amount of non-chromomeric targeting of HA-MeCP2 with its detection on fibrillar extensions that are perpendicular to chromosome axes (Figs. 1 and 4). To test whether these lateral extensions correspond to the bases of the transcriptionally active loops, we co-localized HA-MeCP2 and the protein NF7, a general RNP protein used here as an endogenous marker of most LBC loops, on nuclear spreads (Fig. 4; panel a). Upon careful examination, we found an extensive co-localization of both proteins at the base of many of the LBC loops. Magnified views highlight the fact that while NF7 is present over the entire length of these loops, in association with the RNP matrix, HA-MeCP2 is restricted to a very short domain, at one of the two bases of the loops. This observation is further supported by the lack of the lateral fibrils and smoother appearance of chromomeric HA-MeCP2 distribution pattern when transcription is inhibited with Actinomycin D (AMD) (Fig. 4; panel b). Indeed, the major effects of AMD on the physiology of LBCs are well known and correspond to the retraction of the lateral loops and condensation of the chromosomal axes.

### Immunolocalization of 5-methylcytosine in *Xenopus* and axolotl LBCs

**Fig. 5:**
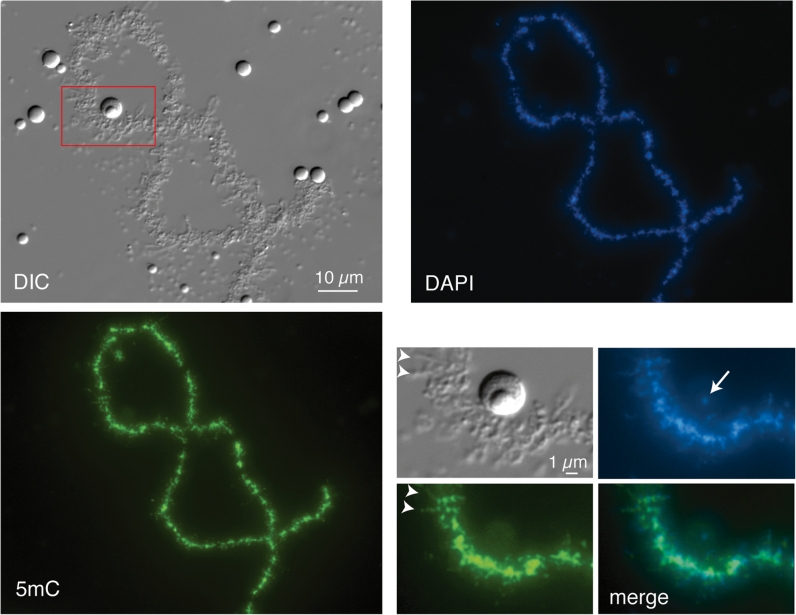
Immunostaining of *Xenopus laevis* LBC with 5-methylcytosine mAb. At lower magnifications a general chromomeric staining for 5mC is evident that is proportional to the DNA concentration indicated by DAPI staining. The region shown at higher magnification in the insets is indicated by the *red box* in the DIC image. *Arrowheads* in the insets indicate two of the 5mC-stained fibrils that project laterally from the chromomeric axis and that presumably correspond to the bases of some lateral loops. Note that the amplified rDNA that can be specifically detected in the fibrillar centres of extrachromosomal nucleoli by DAPI staining (*arrow*) appears unstained for 5mC, consistent with the lack of methylation in amplified rDNA determined by biochemical analyses (Dawid et al. 1970)

The HA-MeCP2 distribution pattern in LBCs described above, and particularly its MBD-dependence, clearly predicts a matching distribution of 5mC-modified DNA in lampbrush chromatin and we have tested this by immunostaining. Although an earlier investigation of *Pleurodeles waltlii* (newt) LBCs showed by immunofluorescence, and also indirectly by electron microscopy, that 5mC was present in compact, transcriptionally inactive chromatin (Angelier et al. 1986), we wanted to re-examine *Xenopus* LBCs in this regard for two reasons. First, the accumulation of large amounts of non-genic DNA, which accounts for the huge genomes of urodele amphibians (newts and salamanders), might distort the extent and distribution of 5mC in LBCs. Secondly, the only evidence for 5mC in non-chromomeric domains of *Pleurodeles* was in loop-associated “globules” detected by e.m., which do not resemble the fibrillar projections described above for MeCP2. Using the well-characterized α-5mC mAb, clone 33D3 (Reynaud et al. 1992; Habib et al. 1999), we found intense immunostaining for 5mC that was overwhelmingly localized in the chromomeres of *Xenopus* LBC axes (Fig. 5). As with the MeCP2 distribution, the intensity of chromomeric 5mC immunostaining appeared proportional to the DNA concentration of individual chromomeres shown by DAPI staining (Fig. 5). However, there was also clear evidence of faintly 5mC-stained fibrillar projections extending from the chromomeres and, as with the MeCP2 localization pattern, comparison with DIC or phase contrast images suggested the fibrils could indeed represent the bases of some of the lateral loops (Fig. 5 inset, arrowheads).

**Fig. 6:**
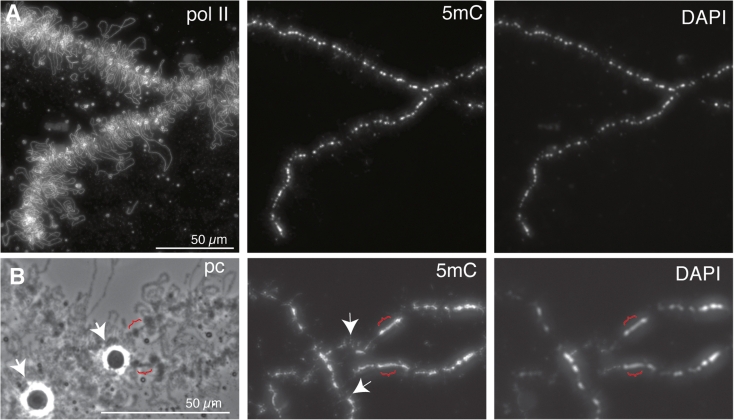
Overview of 5mC-immunostaining of axolotl LBCs. **a** In the portion of a single LBC bivalent shown here a predominantly chromomeric staining pattern that parallels the DAPI staining intensity is apparent under low magnification. The bulk of the loop chromatin, as indicated by pol II (mAb H5) immunostaining appears not to show general 5mC immunostaining. **b** Regions of centromeric heterochromatin are reflected by the appearance of loopless chromatin bars (*brackets*)—the examples here are close to the HLB loci (*arrowheads*) on LBC6. The intensity of 5mC in the heterochromatin appears similar to that of the DAPI staining, as in flanking chromomeres, suggesting that 5mC is not enriched in lampbrush heterochromatin. Note some laterally projecting fibrils of 5mC staining are apparent in neighbouring regions even at this low magnification (two examples indicated by *arrows*)

In order to confirm the origins of the 5mC-containing fibrils we next examined LBC preparations from a salamander, the axolotl *A. mexicanum*. The lateral loops of urodele LBCs are larger, more extended structures than those of *Xenopus*, and so offer the opportunity for more detailed observation of loop structure and function. The loop axes also immunostain particularly brightly using antibodies against the transcriptionally active phosphoisomers of RNA polymerase II (pol II), that are tightly packed along the DNA of active transcription units (Gall et al. 1999). The intense, diffraction-limited line of immunofluorescence produced with pol II antibodies provides a good indication of the precise track of the loop DNA, which due to its low spatial density is otherwise difficult to follow by DAPI staining (Fig. 6; panel a). The overall distribution of 5mC as determined by immunostaining of axolotl LBCs was much as found for *Xenopus*, namely a bright chromomeric immunostaining, roughly proportional to DNA mass shown by DAPI, and a lack of general loop 5mC staining (Fig. 6; panel a). The centromeric regions of axolotl LBCs, which have been mapped cytologically (Callan 1966), appear as loopless bars that are larger and more extended than typical chromomeres and presumably correspond to regions of transcriptionally inactive pericentric heterochromatin. The centromeric heterochromatin clearly did contain 5mC but was not enriched for it, relative to neighbouring chromomeres (Fig. 6; panel b).

**Fig. 7:**
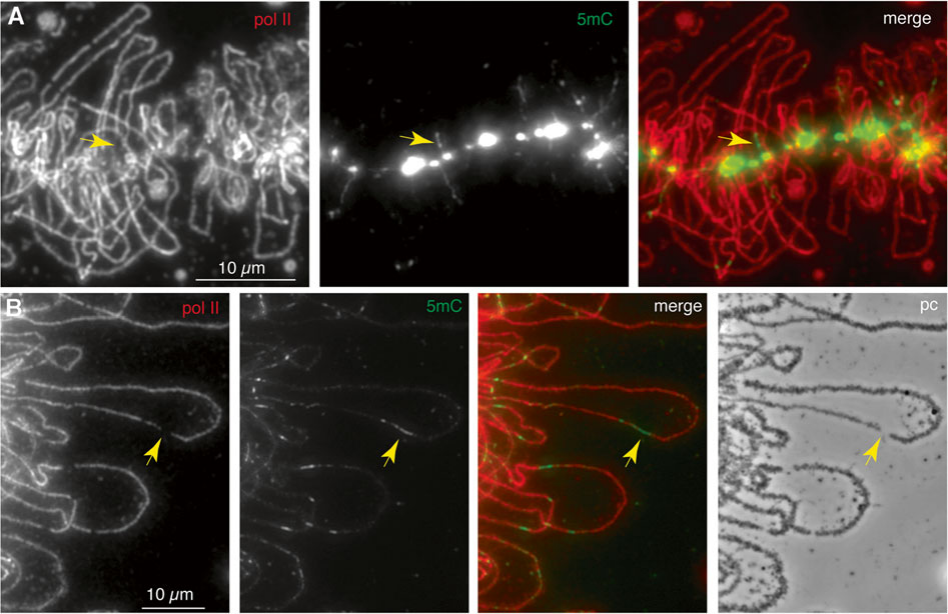
Detail of 5mC-immunostaining of axolotl LBC loops. **a** Laterally projecting 5mC-stained fibrils can be seen emerging from the brightly stained chromomeres of the chromosome axis. The fibrils often appear to be contiguous with, but not to overlap, the transcribed regions of lateral loops that are indicated by pol II staining (mAb H5). One example is indicated by the arrow. **b** Detail of interstitial 5mC immunostaining of lateral loops in axolotl LBCs. Short-stained regions of methylated DNA (*green* in the merge) again appear contiguous with, not to overlap, the pol II-transcribed regions (*red*). Correspondingly, the loop pol II staining pattern exhibits gaps, with the gaps also matching discontinuities in the nascent RNP matrix (phase contrast image). These features are indicated by arrows for one such region in one particular loop, which interestingly appears to comprise a single matrix unit/transcription unit

Closer observation of the chromomeric immunostaining pattern revealed examples of short 5mC-containing lateral fibrils extending for several micrometers from the chromomeric axis as we saw in *Xenopus* LBCs (Fig. 6; panel b). Moreover, after pol II co-immunostaining, there were clear cases in which these fibrils corresponded to the beginnings or ends of certain lateral loops (Fig. 7; panel a). In such examples, it was also apparent that while the 5mC-containing regions could appear contiguous with the pol II-stained regions of the loop, they did not overlap them. It appears then that there may be regions of loop structural domains that can be both non-transcribed and associated with 5mC. This characteristic was shown even more convincingly by short (around 1 μm) segments within the body of a number of loops that were brightly immunostained for 5mC (Fig. 7; panel b). Again, these short interstitial 5mC regions usually appeared interspersed with, but not actually to overlap, the adjacent, extensive pol II-containing regions of the same loop. Moreover, the apparent gaps in the line of pol II staining, with which the 5mC-containing segments corresponded, also appeared to coincide with discontinuities in the array of nascent transcripts that appears in phase contrast images as a matrix of RNP (Fig. 7; panel b, arrows). Lateral loop RNP sometimes gives the appearance of being asymmetrically arranged along the length of a loop, forming a gradient of increasing thickness that corresponds to the elongation of nascent transcripts along the length of an active transcription unit. Since matrix asymmetry identifies both the direction of transcription and the extent of an individual transcription unit, it is possible to conclude for at least some interstitial 5mC-containing regions that they are contained within a single active transcription unit, rather than being non-transcribed “spacers” between two such units (arrowed example in Fig. 7; panel b).

### Immunolocalization of 5-hydroxymethylcytosine in axolotl LBCs

**Fig. 8:**
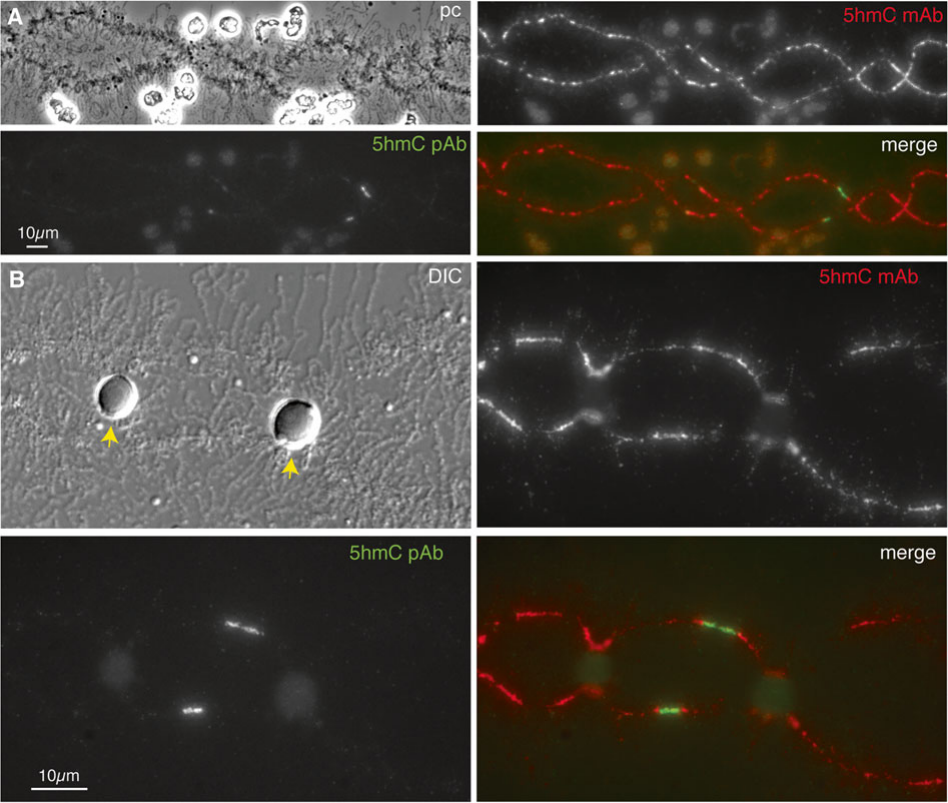
Distribution of 5-hydroxymethylcytosine in axolotl LBCs. **a** Widely distributed immunostaining of chromomeres obtained with a monoclonal antibody against 5hmC in contrast to fewer, more localized foci of immunostaining obtained with a 5hmC polyclonal antibody. Low magnification image of part of LBC3. **b** Central region of LBC13 showing the contrasting patterns of immunolocalization produced by 5hmC monoclonal and polyclonal antibodies. This region of LBC13 contains two loci at which histone locus bodies (HLBs) are always attached, and in this example the HLBs attached at homologous loci are fused to each other (*arrows* in DIC image). A localized region of intense immunostaining with the 5hmC polyclonal antibody occurs about midway between the HLB loci

Although discovered as a DNA modification in T-even bacteriophage many years ago, interest in 5-hydroxymethylated cytosine has increased recently after discoveries of high levels of 5hmC in certain mammalian cell types, including embryonic stem cells (reviewed by Tan and Shi 2012). It appears that 5hmC is produced from 5mC by dioxygenases belonging to the TET family of proteins, and can be further oxidized to 5-formylcytosine and 5-carboxylycytosine by the same enzymes. Since these modified cytosines are recognised by demethylation pathways, it is thought that 5hmC could serve as an intermediate in a process of active demethylation and hence may play a role in the regulation of DNA methylation dynamics (Bhutani et al. 2011). Although further roles for 5hmC in vertebrate genomes are currently the subject of debate, a second function as a stable epigenetic modification in its own right is emerging. One aspect of this is the ability of 5hmC to inhibit MeCP2 from binding methylated DNA and another is the potential recruitment of 5hmC-specific binding proteins (Tahiliani et al. 2009). We felt that the distribution of 5hmC in lampbrush chromatin could be informative in the context of the observations on MeCP2 and 5mC distribution described above. We immunostained axolotl LBCs with two well-characterized, commercially available antibodies that specifically recognize 5hmC; one a mouse mAb, clone 59.1, and the other a rabbit polyclonal antibody. The latter has been shown to prefer densely 5-hydroxymethylated sites to single sites (Stroud et al. 2011). The overall immunostaining pattern we found with the α-5hmC mAb, although less intense, was essentially the same as that found for 5mC; namely a general chromomeric stain proportional to DAPI staining intensity and small fibrillar projections from the chromosome axis representing short segments of loop staining (Fig. 8; panels a and b). The immunostaining pattern produced by the α-5hmC rabbit polyclonal serum was rather different; superimposed on a relatively faint, general chromomeric staining were a small number of intensely stained foci corresponding to short chromomeric regions that were not strongly co-stained by the α-5hmC mAb (Fig. 8) nor by the α-5mC mAb (not shown).

**Fig. 9:**
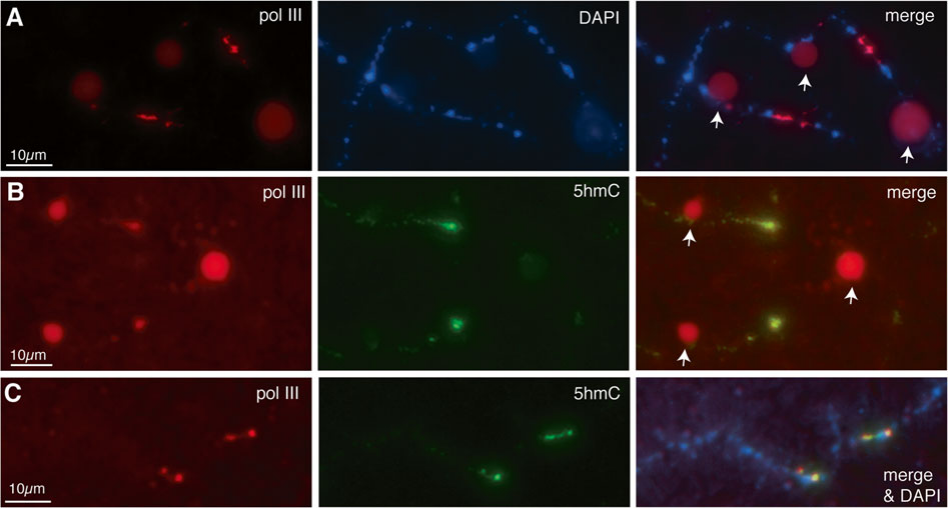
Co-localization of 5hmC and pol III in axolotl LBCs. **a** Central region of axolotl LBC13 immunostained for pol III with α-RPC15 antibody, showing the homologous pol III sites located between the two HLB loci that mark this bivalent. The HLBs at the homologous loci on the left are separate in this example, whereas the HLBs of the right-hand loci are fused (*arrows* in merge). **b** Central region of LBC13 immunostained first for pol III using mAb No34 and then with the α5hmC rabbit polyclonal antibody. The same single region of immunostaining is seen on both homologues with both antibodies. As in (**a**), the HLBs at the left-hand loci are separate while the right-hand HLBs are fused (*arrows* in merge). **c** Two homologous loci from a different LBC in the same preparation as (**b**) also showing co-localization of pol III and 5hmC

Depending on the preparation, there were several to tens of these foci of particularly intense α-5hmC staining spread over the chromosome set, and we mapped some reproducibly to specific locations in the LBCs, such as one in the central region of LBC13 (Fig. 8; panel b). We were able to map these foci by using chromosome-specific morphological features (Callan 1966) and also by an approach developed with *Xenopus* LBCs (Murphy et al. 2002) in which antibodies that recognize RNA polymerase III (pol III) subunits produce unique immunostaining patterns consisting of multiple intensely stained chromosomal sites. There is strong evidence that these chromosomal sites of pol III staining represent regions where transcription of pol III-specific genes is occurring (Murphy et al. 2002). The distinctive morphology of these domains, which is usually as rather amorphous patches rather than the extended lateral loops of pol II transcription units, is thought to be due to the genomic organization of pol III-transcribed genes and the nature of their transcripts. We were struck by the similarity in morphology between pol III sites that we had characterized previously in axolotl LBCs and the foci of intense α-5hmC staining (compare Fig. 8; panel b with Fig. 9; panel a). Moreover some pol III sites mapped to the same location as α-5hmC foci, such as the central region of LBC13 (Fig. 9; panel a). We therefore co-stained preparations using a pol III mAb and the α-5hmC polyclonal Ab and found that indeed, sites of intense 5hmC staining were coincident with pol III transcriptional domains (Fig. 9; panels b and c).

## Discussion

Overall, we have shown that MeCP2 and the 5mC-modified DNA to which it binds are detected primarily in the compact, transcriptionally inactive structural domains of lampbrush chromatin, the chromomeres. However, we have also found evidence that both may also be associated with the extended, active domains exemplified by the pol II-transcribed lateral loops. Moreover, we have also found that there is an apparent enrichment of the oxidized derivative, 5hmC in a different active domain, namely the less extended one occupied by pol III-transcribed genes. What might be the significance of these patterns in the context of this distinctive type of chromatin that typifies amphibian oocytes?

### MeCP2 and 5mC in chromomeres

Although the distribution of the endogenous MeCP2 was not defined here for the lack of a specific anti-*Xenopus* MeCP2 targeting antibody, the association of newly expressed HA-MeCP2 with LBCs, primarily with the more condensed chromomeric regions, is unlikely to be a fortuitous distribution pattern or based on protein–protein interactions. Rather, the association of MeCP2 with LBCs is likely to reflect a direct interaction with methylated DNA. Several lines of evidence support this conclusion. First, we showed that MBD behaves as an autonomous protein domain that is necessary and sufficient for targeting LBCs. Second, two distinct mutations, R106W and R133C, which have their binding affinities for methylated DNA reduced more than 100-fold (Ballestar et al. 2000), fail to associate with LBCs. It is not clear at the moment why a similar mutation, F155S, has little or no effect on the chromosomal distribution of MeCP2, but this discrepancy may simply reflect the structural difference between LBC chromatin and the DNA used to characterize F155S (Ballestar et al. 2000). Finally, the chromosomal pattern of 5mC and HA-MeCP2 are very similar.

There is abundant experimental evidence in support of a model in which MeCP2 represses transcription through binding to methylated DNA and recruiting histone deacetylases to modify the structure of chromatin (Jones et al. 1998; Nan et al. 1998). However, this is not the only mechanism by which MeCP2 may act as a dynamic transcriptional repressor, as it does not always require deacetylase activity (Nan et al. 1998; Kaludov and Wolffe 2000; Yu et al. 2000). In addition, MeCP2 may also act directly by preventing the formation of the transcriptional pre-initiation complex, likely through direct interaction with the basal transcription factor TFIIB (Kaludov and Wolffe 2000). Predictably then, we find that a newly expressed HA-MeCP2 interacts primarily with the condensed and transcriptionally inactive chromomeric regions of LBCs. Furthermore, the fact that the MBD alone associates with the chromomeres further supports the paradigm of an initial recruitment of MeCP2 followed by repression of transcription. Interestingly, in addition to being a repressor MeCP2 may also play an active role in regulating the architecture of silenced chromatin (Buhrmester et al. 1995; Weitzel et al. 1997; Andrulis et al. 1998; Horike et al. 2005; Singh et al. 2008). In fact, MeCP2 is part of a wider network of competing and co-operating chromatin binding proteins that constantly remodel nucleosomes and modulate the accessibility of chromatin fibres. This network includes HMGs A,B and N, UBF, and histone H1 (Bustin et al. 2005). Recently, MeCP2 was shown to bind methylated linker DNA in nucleosome arrays both in vivo and in vitro (Ishibashi et al. 2008) in a similar way to linker histones.

Regardless of MeCP2 roles within chromomeres, its rapid and efficient association with LBCs (it can be detected on LBCs as early as 1 h after injection of its corresponding transcript) indicates a very dynamic nature of its interactions with chromatin. This is in good agreement with the recent demonstration by fluorescence recovery after photo-bleaching that the interaction of MeCP2 with somatic chromatin is very transient (Kumar et al. 2008). It is noteworthy to recall here that LBCs lack histone H1 and the H1-like B4 protein (Smith et al. 1988; Hock et al. 1993). Thus, another possibility for explaining the efficient chromomeric targeting of HA-MeCP2 is a lack of a binding competition from histone H1 and variants. Interestingly, a HA-tagged histone H1 readily targets the chromomeric regions of LBCs, but not the lateral loops (Joseph Gall, personal communication). Clearly, more work will have to be carried out on LBCs to test whether endogenous MeCP2 is present on LBCs, can be exchanged for newly expressed HA-MeCP2 and whether its binding properties are affected by the presence of histone H1.

### The association of MeCP2 and 5mC with lampbrush loops

Perhaps, one of our most exciting findings is the fact that both MeCP2 and 5mC can also be associated with the bases of extended lateral loops. Indeed, this distribution pattern is in very good agreement with the recent finding that more than 60 % of MeCP2 bound promoters in mammalian neurons belong to actively expressed genes (Yasui et al. 2007). In addition, while the expression level of thousands of genes in the hypothalamus of mice is affected by a dysfunction of MeCP2, it was found that more than 80 % of these genes appear to be activated by MeCP2 (Chahrour et al. 2008). Thus, the association of MeCP2 with the base of the loops could represent a population of the protein implicated in transcriptional activation, which would directly support the emerging new model presenting MeCP2 as both a transcriptional repressor and activator.

In some cases in which the long loops of axolotl LBCs allow a detailed observation of their bases, it appears that they are also non-transcribed, so the only distinction with the neighbouring chromomeric chromatin to which the loop is attached seems to be that these methylated regions are extended rather than compacted. Indeed it is feasible that the MeCP2-containing loop chromatin actually represent regions that normally lie on the surface of chromomeric domains and are then subject to disruption during preparation. A well-known feature of LBC preparations is the occasional formation by mechanical disruption of structures known as double-loop bridges, in which a break in the chromomeric axis is spanned by a pair of lateral loops (Callan 1986; see Macgregor *ibid*.). Perhaps the fibrillar extensions of MeCP2 and 5mC-containing loop bases represent a less severe stage of chromomeric disruption? On the other hand, another distinctive property of chromatin at the points at which loops insert into chromomeres has been described that concerns the presence of acetylated histone H4 (Sommerville et al. 1993). Antibodies against particular acetylated isoforms of H4 were found to produce intensely immunostained foci within the masses of chromomeric chromatin and these foci were generally located at points where the lateral loops were anchored. This type of chromatin mark is thought typically to be associated with active domains and its presence in chromomeres was considered unexpected (Sommerville et al. 1993). It seems possible that the somewhat paradoxical associations of MeCP2 and 5mC with loop bases and of acetylated H4 with chromomeric insertion points indicate that the chromatin states of the border regions between these structural domains are in flux and reflect a particularly dynamic region of lampbrush chromatin.

Although at this stage it is difficult to rule out the presence of 5mC in loop bases as arising from mild disruption of chromomeres during preparation, this does not account for the presence in axolotl LBC loops of interstitial segments containing 5mC. At the least, these regions show unambiguously that methylated DNA can exist in the extended, transcriptionally hyperactive structural domains represented by lateral loops. There seem several possible ways to explain the existence of interstitial methylated regions in loops, which may also be applicable to the similar regions at loop bases. One obvious explanation is that these regions represent methylated non-transcribed spacers separating multiple transcription units within a single loop domain. However, as discussed above in relation to Fig. 7; panel b, this is unlikely to be the general case for interstitial loop methylation. Similarly, the explanation that these regions of localized methylation simply correspond to densely methylated intragenic CpG islands as described within transcribed mammalian genes (Jones 2012), including those of oocytes (Smallwood et al. 2011), also appears incomplete. This is because, surprisingly, the interstitial 5mC regions of loops actually seem to be simultaneously non-transcribed (i.e., they are coincident with pol II- and nascent transcript gaps within transcription units that are otherwise maximally packed with transcription complexes). However, the precise correspondence of 5mC-stained loop regions with these gaps suggests that selective detection could provide a simple technical explanation for localized immunostaining. If loop DNA were entirely methylated then 5mC might only be detectable by immunostaining in gap regions because of the increased antibody accessibility resulting from the absence of pol II transcription complexes that normally mask the underlying DNA template. Detection of DNA in transcript gap regions could also be enhanced because such regions would be expected to form more condensed, nucleosomal chromatin (Scheer 1978, 1987). Interestingly, in recent studies assessing the presence of histone H4 in loop chromatin by immunostaining, discrete regions of loop H4 immunostaining also coincided with gaps in the pol II array and the nascent RNP matrix (Fig. 1 in Austin et al. 2009; Fig. 2 in Gaginskaya et al. 2009). Again, it could be argued that the localized immunostaining arises from the enhanced detectability of H4 in the transcript gaps in which DNP targets are not occluded by densely packed pol II. However, it is difficult to distinguish this kind of technical explanation for interstitial loop methylation from an alternative in vivo one; namely that 5mC occurs only in those internal regions of active genes in which a temporary absence of pol II provides opportunistic access for de novo DNA methylation. To distinguish these explanations, it will be important to assess whether the majority of loop DNA is in fact completely modified such that unmethylated CpG dinucleotides are typically absent from regions downstream of active promoters.

Regardless of loop methylation, a second question relevant to loop function in general concerns the significance of the gaps in the pol II and transcript arrays themselves. Simple technical explanations, such as their representing occasional physical breakage or extreme stretching of loops brought about during preparation, are difficult to sustain because 5mC- and histone H4-immunostaining demonstrate that chromatin is clearly detectable in the gaps. It is arguable that the selective stripping of pol II and attached nascent RNP from highly localized, short regions of loops during preparation could be responsible for the gaps revealed by pol II immunostaining. However, this seems unlikely given the stability of pol II attachment observed repeatedly in many investigations using “Miller-spreading” (Miller and Hamkalo 1972), which subjects LBCs to more disruptive preparative conditions than does preparation for light microscopy. Moreover, the attachment of polymerases to loop DNA in Miller spreads, unlike that of nucleosomes, is resistant even to treatment with detergents such as Sarkosyl (Scheer 1978). It seems more likely that the gaps in pol II immunostaining and nascent transcripts observable within loop transcription units are caused by a stochastic feature(s) of steady-state oocyte transcription. Whatever this feature might be, the gaps in the polymerase stream that result are presumably transient; otherwise, production of full-length transcripts by the affected transcription units would rapidly be shut off. The existence of stochastic fluctuations in transcription rate should be particularly amenable to study using lampbrush loop chromatin because their effects on polymerase packing density can be frozen at an instant in time and analysed directly in single transcription units.

### Detection of 5hmC in oocyte chromatin

Immunostaining with two different antibodies against 5hmC provided clear evidence for the presence of this novel modified base in oocyte chromosomal DNA, although the detailed immunostaining patterns were distinctive for each antibody. The 5hmC mAb produced a widely distributed and homogeneous chromosomal staining pattern that was indistinguishable in detail from that of the 5mC mAb. This similarity could indicate a close intermingling of the two types of modified residues within localized regions of the genome and, given the potential role for hmC as an intermediate in the demethylation of 5mC, could reflect the existence of ongoing dynamic processes of de novo methylation and demethylation in the oocyte nucleus. Alternatively, the relatively coarse level of resolution provided by LBC staining might simply mean that at the molecular level 5hmC and 5mC are stably targeted to different DNA sequences even within a given co-stained chromosomal domain such as an individual chromomere, which contains on average about 5 Mb DNA.

By contrast, immunostaining with the 5hmC polyclonal antibody revealed a relatively small number of intensely stained foci on the chromosome axes. We think this distinctive pattern is at least partly explained by the known preference of the polyclonal antibody for multiple clustered 5hmC sites rather than isolated hydroxymethylated sites (Stroud et al. 2011). This explanation is suggested by the other notable feature of the 5hmC foci, namely that many coincide with sites of pol III transcription. Genes transcribed by pol III produce short non-coding RNAs such as 5S rRNA and tRNA (reviewed by White 2011) and typically occur in families composed of tandemly repeated units that in urodeles can be huge in number: in the newt *Pleurodeles* there are about 50,000 5S genes and 150,000 tRNA genes per haploid genome (Van den Eynde et al. 1989). Even though highly repeated, the 5S and tRNA genes of urodele genomes tend not to be highly dispersed but clustered at small numbers of chromosomal loci—for instance, there are thought to be about 75,000 5S genes at each of four loci in another newt, *Notophthalmus viridescens* (Pukkila 1975). Since each pol III gene has its own internal promoter, genomic regions containing these gene clusters can exhibit high densities of the dinucleotide CpG, the predominant target site for 5mC, and consequently for production of 5hmC (e.g. there are six CpGs in each 120 bp axolotl 5S gene, Morgan unpublished). Moreover, despite their high degree of transcriptional activity in oocytes, pol III transcriptional domains appear rather compact compared to the extended pol II-transcribed lateral loops. The presence at lampbrush pol III loci of an extraordinarily high spatial density of the sites preferentially recognised by the hmC polyclonal antibody could therefore be responsible for the apparent enrichment for 5hmC at these loci that is suggested by intense immunostaining. On the other hand, we did not detect foci of intense immunostaining with the polyclonal 5hmC antibody near the expected locations of tandemly repeated genes transcribed by other polymerases, namely the chromosomal rRNA genes at the nucleolus organizer and histone genes at HLB loci (Fig. 8; panel b). Since these tandem arrays might also be expected to produce a high chromosomal density of potential 5hmC sites, there could be an added functional significance to the presence of 5hmC in oocyte chromatin that is specific to pol III-transcribed genes.

A distinctive feature of the developmental regulation of amphibian pol III-transcribed genes was initially discovered for the 5S rRNA genes of *X. laevis* (reviewed by Wolffe and Brown 1988). *Xenopus* produces two major types of 5S RNA, a somatic type found in most cells and an oocyte-type synthesized and accumulated almost exclusively in oocytes. Each type of 5S RNA is transcribed from distinct genes, one comprising a large multigene family of oocyte 5S genes, and the other a much smaller family of somatic 5S genes. The two gene families exhibit contrasting patterns of expression, with oocyte genes becoming activated in oocytes and repressed in the somatic cells of the developing embryo, while somatic 5S genes are active in all cell types. Similar dual gene systems and patterns of expression have since been found for tRNA genes in *Xenopus* (Stutz et al. 1989) and the 5S genes of *Pleurodeles* (Van den Eynde et al. 1989). The establishment and propagation of the repressed state in *Xenopus* oocyte 5S genes during embryonic development was one of the earliest examples of an epigenetic mechanism to be recognised at a molecular level and factors contributing to the differential regulation of the two 5S gene families have been intensively investigated (Wolffe and Brown 1988; Bouvet et al. 1994; Howe et al. 1998). *Xenopus* oocyte-type 5S genes are thought to exhibit high levels of 5mC in somatic cells (Miller et al. 1978). This raises the possibility that production of the high-density 5hmC sites we detected in the pol III structural domains of axolotl lampbrush chromatin is part of a mechanism that marks oocyte-type, pol III-transcribed genes for transcriptional activation in oocytes and subsequent developmental repression in somatic cells.

## Abbreviations

5mC: 5-Methylcytosine
5hmC: 5-Hydroxymethylcytosine
AMD: Actinomycin D
CCD: Charge-coupled device
DAPI: 4′,6-Diamidino-2-phenylindole
Fc: Fragment-crystallizable region
GV: Germinal vesicle
HA: Human influenza hemagglutinin epitope tag
HLB: Histone locus body
IgG: Immunoglobulin G
IgM: Immunoglobulin M
LBC: Lampbrush chromosome
mAb H5: Monoclonal antibody H5
MBD: 5-Methylcytosine binding domain
MeCP2: Methyl-CpG-binding protein 2
NF7: Nuclear factor 7
NLS: Nuclear localization signal
ORF: Open reading frame
PBS: Phosphate-buffered saline
PCR: Polymerase chain reaction
pol II: RNA polymerase II
pol III: RNA polymerase III
RNP: Ribonucleoprotein
SP6: Bacteriophage SP6
